# Biotechnological Advances in Resveratrol Production and its Chemical Diversity

**DOI:** 10.3390/molecules24142571

**Published:** 2019-07-15

**Authors:** Samir Bahadur Thapa, Ramesh Prasad Pandey, Yong Il Park, Jae Kyung Sohng

**Affiliations:** 1 Department of Life Science and Biochemical Engineering, Sun Moon University, Chungnam 31460, Korea; 2 Department of Pharmaceutical Engineering and Biotechnology, Sun Moon University, Chungnam 31460, Korea; 3 Department of Biotechnology, The Catholic University of Korea, Bucheon, Gyeonggi-do 14662, Korea

**Keywords:** resveratrol, metabolic engineering, microbial production, chemical diversity

## Abstract

The very well-known bioactive natural product, resveratrol (3,5,4′-trihydroxystilbene), is a highly studied secondary metabolite produced by several plants, particularly grapes, passion fruit, white tea, and berries. It is in high demand not only because of its wide range of biological activities against various kinds of cardiovascular and nerve-related diseases, but also as important ingredients in pharmaceuticals and nutritional supplements. Due to its very low content in plants, multi-step isolation and purification processes, and environmental and chemical hazards issues, resveratrol extraction from plants is difficult, time consuming, impracticable, and unsustainable. Therefore, microbial hosts, such as *Escherichia coli*, *Saccharomyces cerevisiae*, and *Corynebacterium glutamicum*, are commonly used as an alternative production source by improvising resveratrol biosynthetic genes in them. The biosynthesis genes are rewired applying combinatorial biosynthetic systems, including metabolic engineering and synthetic biology, while optimizing the various production processes. The native biosynthesis of resveratrol is not present in microbes, which are easy to manipulate genetically, so the use of microbial hosts is increasing these days. This review will mainly focus on the recent biotechnological advances for the production of resveratrol, including the various strategies used to produce its chemically diverse derivatives.

## 1. Introduction

**Figure 1:**
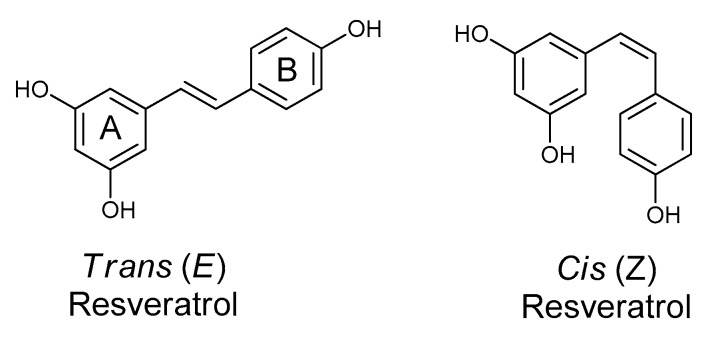
Structures of *trans*-resveratrol and its *cis*-isomer.

Resveratrol is a well-known polyphenol produced as a secondary metabolite by plants [1]. Chemically, it is called 3,5,4′-trihydroxystilbene [2]. According to the International Union of Pure and Applied Chemistry (IUPAC)nomenclature, resveratrol is named 5-[(E)-2-(4-hydroxyphenyl) ethenyl] benzene-1,3-diol. As its basic structure contains a double styrene bond between two phenolic rings, resveratrol exists as *trans-* and *cis*- forms in nature (Figure 1). Among them, the *trans*-isomer of resveratrol has many benefits and is most stable in terms of steric hindrance [2,3,4,5]. However, the *trans*-isomer changes into the *cis*-isomer in the presence of ultraviolet light [6]. Though both *cis*- and *trans*- isomers of resveratrol naturally exist in plants and in wines, the *cis*-isomer of resveratrol has not been reported in grapes so far [7,8,9].

Resveratrol has a long history in scientific literature and has been one of the extensively studied plant metabolites. Takaoka used white hellebore lily (*Veratrum grandiflorum*) for the first time to isolate resveratrol during the 1940s [10] and initially it was characterized as a phytoalexin [10,11,12]. Later, it was identified from about seventy different kinds of plants and its products, such as grapes, peanuts, blueberries, and hops. Plants produce it as defense molecules against different microbial infections and drastic surroundings [13,14,15,16]. Resveratrol has been used for human benefits in different health-care products, cosmetics, dietary supplements, and medicines as a cardiovascular, antioxidant, anticancer, and anti-aging agent [2]. Because of its enormous beneficial health effects on human beings, it is also popular as a major component of “French Paradox”.

Generally, the resveratrol content in plants is very limited [13,17,18,19]. However, there are some rich sources of resveratrol, such as the root of *Polygonum cuspidatum*, rhizomes of *Veratrum formosanum*, and leaves of *V. grandiflorum*, which is domestic giant knotweed [20,21,22]. In nature, different derivatives exist, such as *trans*- and *cis*-resveratrol glucosides, dimers (pallidol), trimers (grandiphenol C), *α*-viniferin, and polymers [1].

## 2. Biological Significance in Humans

Resveratrol has many pharmacological activities in humans because of its multiple abilities to affect target cells, cellular physiology, and signals transmitting activities [23,24]. It is reported that resveratrol eliminates free radicals, such as reactive oxygen species (ROS) and reactive nitrogen species (RNS), produced by the metabolism of the body, minimizes possible radical damage to delicate organs, inhibits lipid peroxidation, and increases cholesterol effluxes in the blood, which ultimately improves neurological and cardiovascular activities [4,25,26,27,28,29]. Resveratrol also has been found to display anticancer activities by increasing a transcription factor and regulating the mechanism of expression of small RNA [30,31]. In addition, resveratrol has been reported to be an anti-aging agent, found in some microorganisms like yeast and in fruit flies, mice, and nematodes, by decreasing cAMP phosphodiesterase activities. It also counteracts the major steps of carcinogenesis by having anti-initiation, anti-mutagen, anti-progression, and anti-promotion activities [17,23,32,33,34,35,36]. According to different reports, resveratrol has been found to be effective for type-II diabetes, obesity, atherosclerosis, Alzheimer’s disease, hypertension, and ischemic injury, including various cancers [30,37,38,39,40,41].

### 2.1. Biosynthesis of Resveratrol in Plants

**Figure 2:**
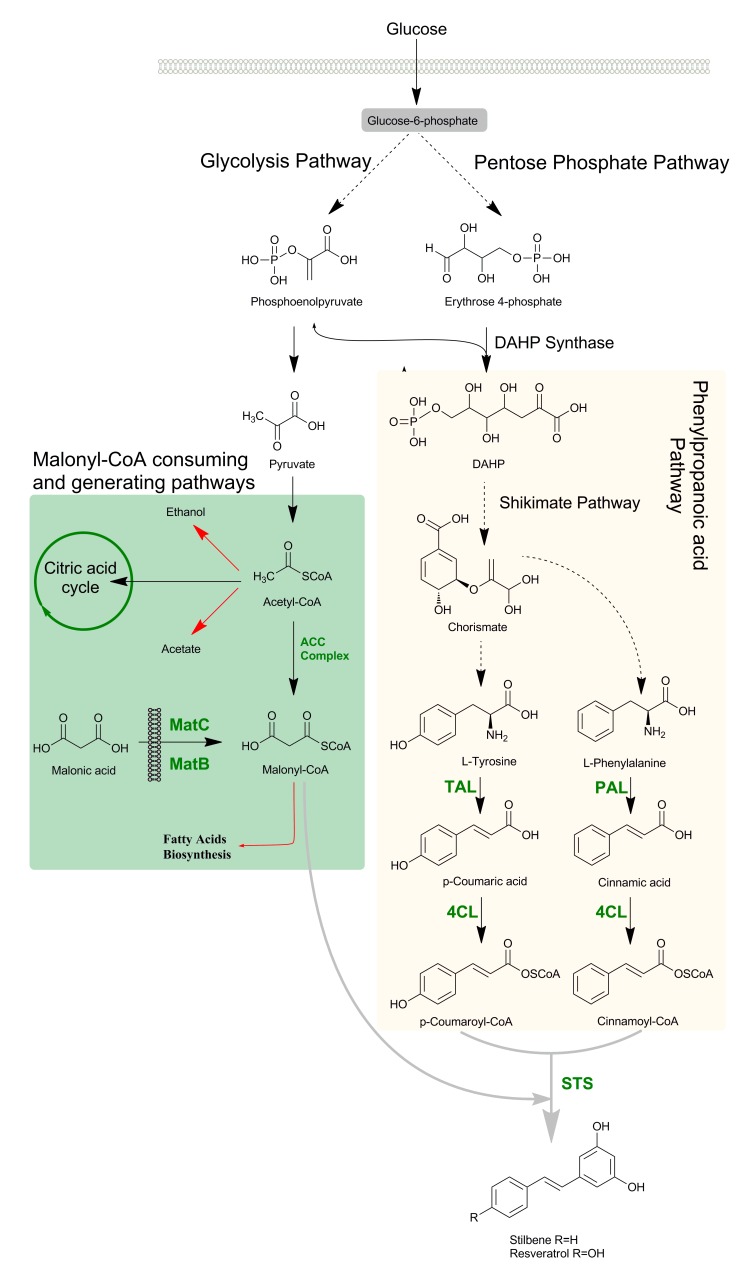
Biosynthesis pathway for resveratrol starting from glucose. The pathways for generating precursors of resveratrol biosynthesis such as phenylpropanoyl-CoAs and malonyl-CoA are highlighted. The pathways consuming malonyl-CoA are shown in red arrows. ACC complex: acetyl-coA carboxylase multienzyme complex; MatB and MatC: malonate assimilating pathway genes; DAHP synthase: 3-Deoxy-D-arabinoheptulosonate 7-phosphate (DAHP) synthase; TAL: tyrosine ammonia-lyase; PAL: phenylalanine ammonia-lyase; 4CL: 4-coumaroyl-coA ligase; STS: stilbene synthase. Dotted arrows indicate multiple steps.

In plants, stilbenes are produced by the universal route of phenylpropanoid, which is the major metabolic pathway. Biosynthesis of phenylpropanoid begins from either phenylalanine or tyrosine obtained from glucose by the Shikimate or Arogenate pathway [42,43] (Figure 2). The biosynthesis of amino acids begins with the enzyme 3-deoxy-D-arabino-heptulosonate-7-phosphate (DAHP) synthase, which helps to condense phosphoenolpyruvate (PEP) and erythrose-4-phosphate (E4P) to generate DAHP [44]. In three isoforms, DAHP synthase occurs in bacteria, which regulate each other by a feedback-inhibition allosteric mechanism [45]. With the help of six enzymes, DAHP is converted into chorismate (CHO), which is the main precursor of L-phenylalanine (L-Phe) and L-tyrosine (L-Tyr) [46]. In most bacteria, such amino acids are the final products of the biosynthetic pathway, but in plants and some bacteria, these amino acids are formed as intermediate products to synthesize secondary metabolites such as phenylpropanoids [44]. During the biosynthesis of resveratrol, first of all, *p*-coumaric acid is formed by either L-Phe or L-Tyr by the removal of ammonia under the action of phenylalanine ammonia lyase (PAL), producing *trans*-cinnamic acid, which is further hydroxylated with the help of cinnamate -4-hydroxylase (C4H) to give *p*-coumaric acid. But by *Zea mays* L, L-Tyr is used to make *p*-coumaric acid with the activation of tyrosine ammonia lyase (TAL) enzymes instead of PAL [47]. Then the *p*-coumaric acid by its esterification with coenzyme A (CoA) is changed into *p*-coumaroyl-CoA by the action of 4-coumaroyl-CoA ligase (4CL). Finally, with the help of stilbene synthase (STS), three molecules of malonyl-CoA are condensed with 4-coumaroyl-CoA through a repeating decarboxylating reaction to cyclize a tetraketide intermediate to produce resveratrol (Figure 2) [47,48,49]. A list of the characterized stilbene synthases is provided in Table 1.

### 2.2. Alternative Sources of Resveratrol

Even though resveratrol is produced naturally in plants, it is very difficult to extract resveratrol in commercial quantities because of its low concentration, multiple steps of isolation and purification, environmentally unfriendly, and seasonal occurrence [58]. However, commercially, resveratrol has been extracted from wild *polygonum cuspidatum’s* root (Japanese knotweed), grape skins and seed (where it has been reported to be between 1.9 and 12.6 mg/L), and the domestic giant knotweed of China, which is the world’s largest producer [20,58,59,60,61]. Therefore, to address the high demand for resveratrol, there is a need for alternative methods because of its high medicinal and dietary value. Here we focus on the trends of producing resveratrol in brief.

A comparatively high amount of resveratrol can be produced by chemical synthesis, but it suffers from the formation of many unwanted side products that contaminate the resveratrol and make purifying it complicated. Thus there is a high risk in using such resveratrol as medicines and food ingredients [62,63,64]. Different biotechnological approaches, such as tissue culture and genetic engineering, have been applied as alternative bio-sustainable resources to produce resveratrol. Callus culture, plant culture, hairy root culture, cell suspensions culture, genetically modified transgenic plants, and recombinant microbes are well-established methods [65]. For a long time, several high-value compounds have been produced successfully in microorganisms [66]. The genetic engineering of the host strain was done to integrate the heterologous pathways from plants with the host strain, and significant success in the bioproduction of resveratrol was achieved [58,67]. After the successful achievement of resveratrol production in yeast by Beekwilder and his team in 2006 for the first time [68], several studies have been done for the scale-up production of resveratrol in different hosts; these have obtained 3.1 to 812 mg/L in yeast [69,70] and 1.4 to 2.34 g/L in bacteria [71,72]. Furthermore, the production of resveratrol is remarkably improved by using metabolic pathway engineering and expressing natural plant pathway genes [14,68]. For the commercial production of resveratrol, recent biotechnological approaches have focused categorically on two strategies, engineering of the biosynthetic route of forming the phenolic **B** ring of resveratrol (stilbenes) and engineering the upstream and downstream pathways for increasing malonyl-CoA, which is condensed by stilbene synthase to make the phenolic **A** ring of the resveratrol [43]) (Figure 1).

### 2.3. Microbial Biosynthesis of Resveratrol

The commonly used hosts for resveratrol production are *Escherichia coli*, *Lactococcus lactis*, *Streptomyces venezuelae*, and *Corynebacterium glutamicum* as prokaryotes and yeast *Saccharomyces cerevisiae* as eukaryotes. Normally, it is very important to select a suitable host organism for the optimization of a high product. There are pros and cons for both bacteria and yeast as a host. Bacterial hosts have a short life cycle, easy genetic manipulation and handling, a higher growth rate, and good ability for protein and enzyme overexpression [73,74], but expression of large proteins and post-translational modifications, which are essential for the correct folding and functional activity of recombinant proteins, are greatly lacking [75]. Whereas yeast (*S. cerevisiae)* has no complications on post-translational modifications, has more expression of membrane proteins, is well characterized, is easier to grow and manipulate, and has a food-grade status (generally recognized as safe (GRAS)organism) it is highly sensitive to use of a high concentration of *p*-coumaric acid, and has a lower yield than bacteria [76,77,78,79].

Metabolic engineering, *de novo* pathway engineering, alternative enzyme selection and protein engineering, system and synthetic biology approaches, and central carbon flux redirection have facilitated bio-production of resveratrol in different microorganisms [80].

### 2.4. Biosynthesis of Resveratrol in Non-E. coli Hosts

*S. cerevisiae* is a popular and ideal GRAS-recognized host for the production of diverse plant-derived molecules that can be easily genetically manipulated [79,81]. After successfully implanting the 4-coumarate coenzyme A ligase (4CL) from *Nicotiana tabacum* and stilbene synthase (STS) from *Vitis vinifera* to the yeast by Beekwilder and his team for the first time, producing 6 mg/L of resveratrol, many researchers have tried inserting two enzymes from different sources, STS and 4CL, to produce resveratrol from the milligram to gram scale in engineered yeast [68,70,82,83,84]. Another yeast strain, *Yarrowia lipolytica*, was recently engineered to produce 1.46 mg/L of resveratrol [85].

Recently, apart from yeast, an engineered strain of *Corynebacterium glutamicum* has become popular for small-molecule production [86,87,88]. The engineered *C. glutamicum* (*ΔphdB*, *ΔpcaF*, and *ΔpobA*) was generated to produce resveratrol from *p*-coumaric acid and produced 158 mg/L of resveratrol when the fatty-acid biosynthesis inhibitor cerulenin was added to the medium [89]. Very recently, Braga and his team designed a system to produce resveratrol successfully in a *C. glutamicum Del*Aro4 strain using glucose while supplying cerulenin, a fatty-acid synthase inhibitor, to increase the pool of malonyl CoA inside the host organism [71,90,91].

*Streptomyces venezuelae* has also been engineered to produce various bioactive natural compounds, including flavonoids and stilbenes. The first report of manipulation of *Streptomyces* sp. was published in 2009 by Park and colleagues, by expressing the heterologous phenylpropanoid biosynthetic pathway genes [92]. They used 4-CL from *S. coelicolor* (ScCCL) and codon-optimized STS from *Arachis hypogaea*. However, the engineered strain produced less than 0.4 mg/L of resveratrol. Similarly, other organisms such as *Aspergillus niger*, *Lactobacillus lactis*, and *A. oryzae*, have also been used for the bioproduction of resveratrol by incorporating the heterologous pathway genes phenylalanine ammonia lyase (PAL), cinnamate-4-hydroxylase (C4H), and 4CL of *Arabidopsis thaliana* and STS of *Rheum tataricum* [93].

### 2.5. Biosynthesis of Resveratrol in E. coli Host

The resveratrol biosynthetic pathway genes are implanted into the *E. coli* host for the heterologous expression to produce resveratrol by metabolic and pathway engineering. During microbial engineering, the heterologous pathway genes from the plants, prokaryotes, and eukaryotes are incorporated inside the useful hosts [94]. Having many advantages, such as short life cycle, high growth rate, easy handling, and comfortable genetic manipulation, *E. coli* is taken as an ideal host for the engineering and production of different biomolecules. Furthermore, successful biotransformation of externally supplied chemicals and *de novo* production of the bioactive compounds using cheap renewable carbon sources are valuable traits in microorganisms [77,95,96]. Through metabolic engineering, the production of resveratrol in *E. coli* has increased by using the precursor tyrosine and malonyl-CoA. So far, all the published reports indicate that the exogenous supply of the precursors, such as tyrosine and *p*-coumaric acid in *E. coli*, is the key to increasing resveratrol production. One major advantage of using *E. coli* rather than yeast as a host is that it can easily tolerate more than 3 g/L of *p*-coumaric acid [70,97]. Additionally, metabolic engineering in the host has demonstrated more opportunities for efficient heterologous gene expression, increasing the pool of precursors, and increasing the amount of intercellular malonyl-CoA necessary for the maintenance of physiochemical conditions of the cell for the higher production of resveratrol [98].

### 2.6. Pathway Engineering

For the production of value-added bioactive resveratrol, pathway engineering is one of the pioneer methods in designing *E. coli*. Given an approximately 50% bioconversion rate, 105 mg/L of resveratrol was obtained from the engineered strain of *E. coli* harboring 4CL from *A. thaliana* and STS from *A. hypogaea* when 1 mM *p*-coumaric acid was used as a precursor [99], whereas about 80.5 mg/L resveratrol was obtained with the same amount of *p*-coumaric acid substrate in *E. coli* implanted with the fusion gene of 4CL and STS from *A. thaliana*, which clearly indicates a lower resveratrol production amount in the fusion genes from the same sources [100]. Similarly, Lim and colleagues made a different combination of 4CL and STS from two *E. coli* strains and obtained 1.3 g/L resveratrol by the strain *E. coli* BW27784. Further, with the addition of cerulenin in the best engineered strain, they obtained 2.3 g/L resveratrol [71]. About 37 mg/L resveratrol was obtained from the *E. coli* strain incorporating PAL of *Rhodotorula rubra*, 4CL of *Lithospermum erythrorhizon*, and STS of *A. hypogaea*, which was more than from the *E. coli* strain having TAL from *R. glutinus*, 4CL of *P. crispum*, and STS of *V. vinifera* [73,101]. But Wang et al. obtained about 114.5 mg/L of resveratrol from the strain harboring TAL from *Saccharothrix espanaensis*, 4CL from *A. thaliana*, and STS from *A. hypogaea* in *E. coli*, which was significantly more [102] than from the existing one. The intracellular pool of tyrosine also plays a crucial role in resveratrol production. The *E. coli* strain engineered by incorporating TAL, 4CL, and STS from *S. espanaensis*, *S. ceolicolor*, and *A. hypogaea*, respectively, produced 1.4 mg/L of resveratrol from tyrosine [72]. Similarly, for the first time, Liu and his team used a site-specific integration strategy for the biosynthesis of resveratrol in *E. coli* [77], where they integrated the genes TAL, 4CL, and STS into the loci of the genes *tyrR* and *tyrRD* in the chromosome of *E. coli* BW25113 (DE3) and obtained 4.612 mg/L of resveratrol (Table 2).

### 2.7. Steps for Increasing the Precursor Pool

As has been mentioned, malonyl-CoA condensation by STS makes the **A** ring of the resveratrol or stilbenes. Malonyl-CoA is a prime precursor of the resveratrol biosynthesis. Naturally most of the malonyl-CoA is used in the fatty-acid biosynthesis. Hence, only a small amount of available malonyl-CoA needs to be used for the resveratrol biosynthesis when heterologous biosynthesis pathway genes are incorporated. Since very little malonyl-CoA is synthesized in *E. coli* [115], it is most important to devise and implant approaches that increase the malonyl-CoA in *E. coli*. For doing so, there are two strategic steps that can be carried out in microbial hosts—(1) inhibition of the fatty-acid biosynthetic gene so that malonyl-CoA consumption pathways are blocked and (2) increasing the carboxylation of acetyl-CoA to make a larger cytosolic malonyl-CoA pool. The overexpression of acetyl-CoA carboxylase (ACC) resulted in a three-fold increase in the cytosolic malonyl-CoA concentration in the *E. coli* host [116]. Furthermore, along with the overexpression of acetate-assimilating enzyme (*acs*) on one side, deleting the competing pathway enzymes encoding genes such as *pta* and *ackA* involved in acetyl-CoA degradation to form acetate and the *adhE* gene involved in ethanol production, showed a 15-fold higher production [117]. There are different reports on the inhibition of fatty-acid biosynthesis that show the efficient production of resveratrol titers. Therefore, there are other approaches as well to blocking the fatty-acid biosynthesis in order to increase intracellular malonyl-CoA. The addition of cerulenin, a covalent inhibitor of *Fab*B and *Fab*F, which are key enzymes in fatty acid biosynthesis, has revealed a significant titer of malonyl-CoA in the host [71,89,117,118]. However, cerulenin is very expensive and also blocks the cellular growth rate of the host itself [119,120,121]. Similarly, the *fab* operon has also been successfully downregulated by using anti-sense RNA in *E. coli* to increase the malonyl-CoA pool, and finally helped to redirect to resveratrol formation. As a result, the operon of the *fab*D gene was inhibited [14] which results in a 4.5-fold increment of the cytosolic malonyl-CoA pool and about 1.5 times more resveratrol production (268 mg/L). Wu and colleagues have successfully downregulated five genes of the *fab* operon (*fabB*, *fabD*, *fabF*, *fabH*, and *fabI*) by using the recently introduced biosynthetic tool CRISPRi and have gotten highly increased resveratrol from 80.0 to 216.5% separately [106]. Additionally, the malonate-assimilating biosynthetic pathway genes (*matB* and *matC*) from *Rhizobium trifolii* were also introduced in the system by Wu and his team to make more cytosolic malonyl-CoA; they obtained a 188.1 mg/L resveratrol titer. Furthermore, the synthetic pathway modified to express TAL by the same team, who successfully obtained 304.5 mg/L resveratrol, can serve as an efficient new synthetic tool in *E. coli* to produce resveratrol despite the production at milligram scale.

### 2.8. Protein Engineering

Sometimes hosts cannot produce enough targeted product because of either poor expression of enzymes or enzymes having a limited turnover. In such a case, by evolutionary or rational engineering methods, the efficiency of enzymes can be improved to make them effective and functional [122]. For higher and more efficient resveratrol production, protein engineering and mutagenesis of 4CL and STS have been done and expressed in *E. coli*. With the thought that co-localization of the two enzymes’ active sites might improve the efficiency, the unnatural fusion of *4CL* from *A. thaliana* and *STS* from *A. hypogaea* was incorporated in *E. coli*, which produced 80.5 mg/L resveratrol when fed 1 mM *p*-coumaric acid [100]. Previously with the similar strategies of introducing resveratrol biosynthesis pathway genes, such as *RsTAL* from *R. shaeroides*, *At4CL* from *A. thaliana*, and *VvSTS* of *V. vinifera* in *S. cerevisiae*, produced only 0.65 mg/L resveratrol starting from *p*-coumaric acid, but with the translational fusion of two proteins, At4CL and VvSTS, produced by substituting the stop codons of At4CL with three amino acids linker into the open reading frame of VvSTS, helped to increase the production to 5.25 mg/L resveratrol, which was almost 3500 times higher than in Becker and his team’s report in 2003. Herein effective channeling of intermediates between proteins or active sites is vital for the higher production of the targeted compound [123]. Similarly, a yeast host harboring codon optimized RsTAL and fused At4CL and VvSTS produced 1.06 mg/L resveratrol without the use of L-Tyr and 1.90 mg/L resveratrol using L-Tyr in 48 h incubation [108]. In a separate study, Wu and colleagues improved the resveratrol production by expressing codon-optimized TAL, 4CL, chalcone synthase (CHS), and CHI (chalcone isomerase) in *E. coli* and obtained 35 mg/L resveratrol [73] (Table 2).

### 2.9. De novo Pathway Engineering

Several aforementioned studies reported the production of resveratrol in the initial precursor substrates, such as systems supplemented with L-Tyr, L-Phe, and *p*-coumaric acid. The use of these precursors in large-scale fermentation reactions is impracticable because of its high market price and possible toxicity in high concentrations [119]. Thus, there is a need to use different approaches of producing resveratrol on a commercial scale by using cheap and sustainable substrates, such as glucose and other carbohydrates [82,106]. To address these problems, a *de novo* pathway approach has been used as a good alternative for the production of resveratrol. In this approach, resveratrol can be produced by using glucose or ethanol instead of highly expensive *p*-coumaric acid in *S. cerevisiae*. By introducing TAL from *Herpetosiphon aurantiacus*, 4CL from *A. thaliana*, and STS from *V. vinifera*, 2.73 ± 0.05 mg/L of resveratrol was produced from glucose. Then overexpressed feedback-insensitive alleles of ARO4, encoding 3-deoxy-D-arabino-heptulosonate-7-phosphate, and ARO7, encoding chorismate mutase, produced 4.85 ± 0.31 mg/L resveratrol. Further, improving the supply of malonyl-CoA by overexpressing a post-translational deregulated version of the acetyl-CoA carboxylase-encoding gene ACC1 helped to increase the resveratrol to 6.39 ± 0.03 mg/L. The strain engineering by integrating multiple pathway genes produced 235.57 ± 7 mg/L, which further produced 415.65 mg/L and 531.41 mg/L resveratrol in fed-batch fermentation from glucose and ethanol, respectively [82] (Table 2).

For the biosynthesis of resveratrol, L-Phe and L-Tyr are very important precursors that are converted into phenylpropanoids by non-oxidative deamination. Therefore, researchers have been focusing on the significant tools that can optimize the production of these aromatic amino acids produced from the shikimate pathway in *E. coli* [78,124,125]. For this purpose, the most commonly used method to increase the flux of L-Tyr and L-Phe biosynthesis is by the removal of enzyme feedback inhibition regulation and transcriptional regulatory processes [126,127]. The multistep systems are devised and implemented in the *E. coli* host to direct carbon flux towards chorismate, a branch point to phenylalanine and tyrosine that can be done by increasing the pool of erythrose-4-phosphate (E4P) and phosphoenolpyruvate (PEP) (Figure 2). The overexpression of PEP synthase encoded with the *ppsA* gene redirects pyruvate to PEP for aromatic-acid biosynthesis. Furthermore, other steps, like the overexpression of transketolases, especially the TKT encoded *tktA* gene, and by repression or disruption of the global regulatory gene *csrA*, can be introduced to increase the aromatic amino-acids pool [80,126,127,128].

The modular rearrangement and optimization of pathway genes, such as RgTAL from *Rhodotorula glutinis*, Pc4CL from *Petroselinum crispum*, VvSTS from *Vitis vinifera*, and RtmatBC from *Rhizobium trifolii* by modular metabolic engineering increased resveratrol production. Previous trends of many studies were more focused on the metabolic pathways connected with resveratrol production, but Zhao and his team focused equally on the intracellular environment of *E. coli* for expressing heterologous genes efficiently and applied a combinatorial optimization strategy. They first experimented by taking the genes *At4CL*, *Sco4Cl* (*S. coelicolor*), *Rs4CL* (*Rhodobacter shaeroides*), *VvSTS*, and *AhSTS* and analyzed the appropriate combination and expression pattern to biosynthesize resveratrol using *p*-coumaric acid. Then, they investigated the intracellular pool of malonyl-CoA by redirecting the pathway of increasing malonyl-CoA by inhibiting the fatty-acid biosynthetic pathway using synthetic sRNA to repress *fabD* expression and increasing malonyl-CoA by expressing *CgaccBC-CgdtsR1*. They had observed that a lower copy number expression of VvSTS and a higher copy number expression of At4CL produced the highest resveratrol [129].

The higher accumulation of resveratrol inside the cell could have negative cellular effects and decrease resveratrol biosynthesis in the cell. Therefore, to balance the intracellular content and produce the higher amount of resveratrol, the resveratrol produced inside the cell was taken out from the cell and immediately transported outside using the four types of endogenous transport systems of *E. coli*. There are six multidrug efflux pump proteins, such as MdfA, EmrD, EmrE, AcrAB, TolC, and MarA, two YddG for amino acids and AraE for L-arabinose transporters, and two OmpW and OmpF outer membrane proteins; these ten proteins were selected by Zhao and colleagues, who did a thorough investigation of their effect in an engineered *E. coli.* The results were compared by overexpressing different transporter genes and resveratrol production. The overexpression of MarA gave 123.16 mg/L and of OmpF gave 151.89 mg/L of resveratrol, which was the highest production of resveratrol among all of the ten transporter proteins. Besides these, they also expressed chaperones *GroES* and *GroEL*, finally combined all four strategies, and got 234.71 mg/L resveratrol [129] (Table 2).

### 2.10. Central Carbon-Flux Redirection

The major challenge of resveratrol production in engineered microorganisms is the low availability of malonyl-CoA. Therefore, most studies focus on how to enlarge the malonyl-CoA pool in the host intracellular space. Lim and his team produced 1308 mg/L resveratrol, whereas Wu and colleagues got 35 mg/L resveratrol in *E. coli* strains by feeding *p*-coumaric acid and L-Tyr, respectively [71,73]. However, the analysis of the cultured sample showed no complete conversion of externally supplied precursors into products [89,101,130,131] because there were too little of the malonyl-CoA essential for STS to form resveratrol [132]. In the normal cellular state, malonyl-CoA is used for the biosynthesis of fatty acids and phospholipids in microorganisms, which ultimately decreases the cytosolic malonyl-CoA pool and lowers the production of secondary metabolites [115]. There must be enough malonyl-CoA for normal cell growth as well as resveratrol biosynthesis. So different strategies have been employed to enlarge the malonyl-CoA pool for the increased production of resveratrol [71,89,117,118]. Gaspar and colleagues demonstrated a four-fold increase in resveratrol by adding cerulenium in *L. lactis* [109]. Similarly, Lim *et al.* also observed 2.3 g/L resveratrol by a two-step biotransformation with feeding *p*-coumaric acid and cerulenin in the *E. coli* strain harboring 4CL from *A. thaliana* and STS from *V. vinifera* [71]. But practically, as said earlier, not only is cerulenin very expensive but a high concentration of it hinders cell growth, so large-scale use of it is inappropriate [71,91,120,131]. There are other strategies as well that focus on rerouting the endogenous malonyl-CoA pathway flow for the stoichiometric modeling, resulting in a higher resveratrol production by increasing the malonyl-CoA pool [133]. Besides these, there are other strategies as aforementioned, such as inhibiting the *fab* operon using antisense RNA [14,129] and downregulating the expression of fatty-acid biosynthesis by using the CRISPRi system [65], and overexpressing of ACC, *acs* (acetate assimilation enzyme) [134] along with the deletion of *pta*, *ackA*, and *adhE* [96] which helps to increase the cytosolic malonyl-CoA pool. Moreover, malonyl-CoA is also increased by overexpressing the *matB* and *matC* genes encoding malonate-CoA synthase and malonate carrier proteins, respectively [134,135]. Recently, a modular engineering approach was used to increase the pool of malonyl-CoA by overexpressing acetate as well as malonate assimilation pathway genes from three different sources. Along with 4CL, STS, and HpaBC enzymes encoding genes, significant amounts of resveratrol and its hydroxylated derivative piceatannol were produced [134].

### 2.11. Optimized Conditions for Higher Resveratrol Production

Some of the significant studies carried out for the optimization of resveratrol production are worth applying for higher resveratrol production in order to meet the world’s demand in a sustainable way. Braga and colleagues have demonstrated the significance of cultivation conditions, such as substrate concentration, on resveratrol production and observed 4 mg/L to 12 mg/L resveratrol on increasing glucose concentration from 40 to 80 g/L, respectively, in *C. glutamicum* [136]. Similarly, in another study, the concentration of dissolved oxygen was also found to be vital for cell growth and resveratrol production, because having more oxygen may oxidize the resveratrol [136,137,138]. Braga et al. (2018b) also observed that a greater oxygen supply in a bioreactor results in less production of resveratrol with *C. glutamicum* [136]. Because of the high metabolic burden in *E. coli*, greater production of resveratrol is not possible despite optimum effort, as was sorted out by Zhou [139], who demonstrated a co-culture system where the whole pathway was divided and introduced in separate strains. Recently, Camacho-Zaragoza et al. (2016) [49] have obtained 22.6 mg/L resveratrol using a glycerol carbon source by the same strategy of co-culturing. There are other many hurdles to making resveratrol in microbial hosts, such as the use of expensive precursors, the toxicity of precursors, and resveratrol itself, which were successfully overcome by using engineered strains that use easily available cost-effective and sustainable substrates, such as glucose [82,106] and by using a fed-batch culture to mitigate the toxicity of precursors [97,99,125]. The toxicity of higher concentrations of resveratrol was addressed by applying in situ product removal from the fermentation broth, facilitating transporter proteins, and regulating the downstream processing [129,140]. Braga and colleagues (2018) applied an in situ removal method with *C. glutamicum* by using Amberlite XAD-7HD as the adsorbent and got 75 to 95% higher resveratrol production [136].

### 2.12. Microbial Production of Resveratrol Derivatives

**Figure 3:**
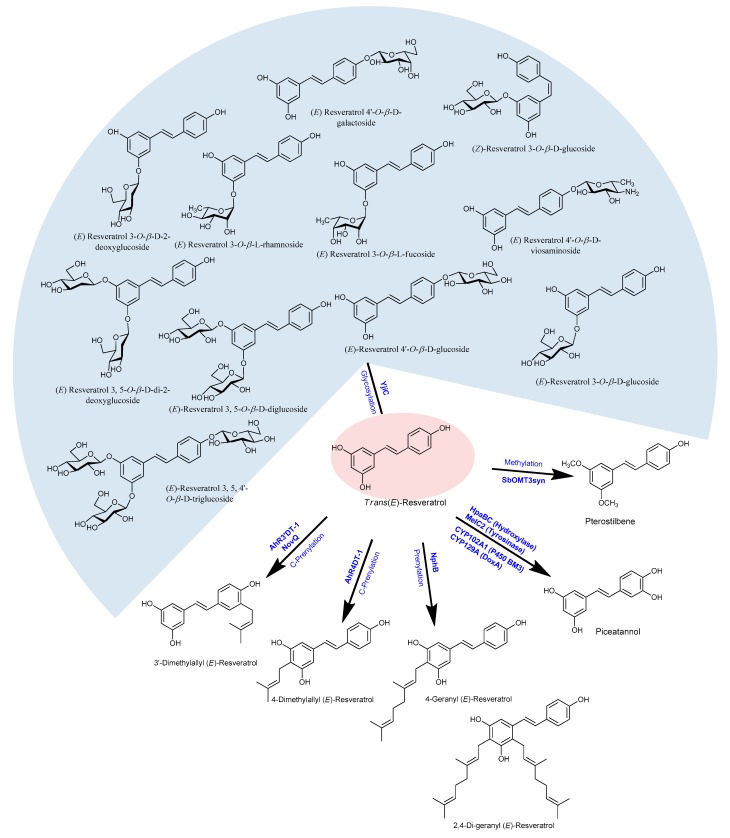
Structures of resveratrol derivatives produced using different post-modification enzymes.

Since the backbone of all stilbenes is based on the 14-carbon skeleton with two phenyl rings connected by an ethylene linker, the biosynthetic pathway of the basic structure of stilbenes is the same as that of resveratrol. Because resveratrol and piceatannol have shown the highest cancer chemopreventive activities, recent research trends are shifting on the derivatives of resveratrol (including its oligomers). Some investigations have demonstrated that the derivatives of resveratrol had shown higher antioxidant activities than did resveratrol [141,142]. Because of not only its high medicinal and nutritional values but also its higher stability against light, oxygen, and extreme *p*H than resveratrol, the production of derivatives of resveratrol is increasing in demanded day by day. Moreover, structure-activity studies have shown that the substitution of methoxy groups for the hydroxyl groups in resveratrol significantly increases its cytotoxic activity [143]. Some of the natural and modified derivatives of resveratrol are pterostilbene, piceatannol, *trans*-piceid, *trans*-ε-viniferin, arachidin-3, gnetol, pinosylvin, rhapontin, oxyresveratrol, astringin, rhapontigenin, isorhapontigenin, pinostilbene, trimethoxy-stilbene, dihydroxystilbene, resveratrol-triacatate, dialloylresveratrol, and pallidol. The modified derivatives of resveratrol are produced by glycosylation, methylation, oligomerization, isomerization, halogenation, or isoprenylation [144,145,146,147] (Figure 3).

Methylated resveratrol derivatives, namely, pinostibene and pterostilbene, were produced in recombinant *E. coli* having SbROMT3syn using resveratrol as the substrate [111]. The same team further expressed the codon-optimized resveratrol biosynthesis genes, such as *ScCCL* (Cinnamate/ 4-coumarate—coenzyme A ligase from *S. coelicolor*), *RpSTSsyn* (stilbene synthase from *Rhubarb (Rheum) species*), and *SbROMT3sysn* in *E. coli*, and successfully produced methylated derivatives of resveratrol using *p*-coumaric acid [148]. A similar type of methylated derivative of resveratrol was also produced by Kang and colleagues by assembling codon-optimized *S. espanaesis TAL*, two *O*-methyltransferase (*SbOMT1* and *SbOMT3*) from *Sorghum bicolor*, and a codon-optimized *AhSTS* and *ScCCl* under T7 promoter in *E. coli* [149]. Moreover, the additional expression of the glycosyltransferase gene *YjiC* (from *Bacillus licheniformis* DSM13) to the resveratrol biosynthesis monocistronic pathway produced 7.5 mg/L resveratrol-4′-*O*-glucoside, 2.5 mg/L trans-resveratrol-3-*O*-glucoside (*trans*-piceid), and 1.7 mg/L *cis*-resveratrol-3-*O*-glucoside [1,110]. In a different approach of *in vitro* enzymatic glycosylation, ten different derivatives of resveratrol glycosides were produced [1].

Similarly, a hydroxylated product of resveratrol called piceatannol is biosynthesized in some of the plants because of the action of pinosylvin synthase, since it was suspected for its multifunctional activities [52]. About 55 mg/L of piceatannol was isolated from *C. glutamicum*, starting from 5 mM caeffic acid added in the medium in the presence of 25 µl cerulenin [71,90,92]. About 1.2 g/L piceatannol was obtained in *E. coli* using non-P450 hydroxylase *hpaBC* with a resveratrol substrate [150]. Furthermore, 5.2 g/L piceatannol was reported by Furuya and Kino (2014) in the presence of Tween 80 in whole-cell catalysis with *hpaBC* monooxygenase [151]. Similarly, Rimal et al. (2018) have also made piceatannol by the hydroxylation of resveratrol using DoxA, a *Streptomyces peucetius* CYP450 enzyme, in the presence of ferredoxin reductase and ferredoxin [152].

Furthermore, Yang and coworkers (2017) have observed the C-prenylation on resveratrol or stilbenoids at the 4- and 3′-position by the help of the enzymes AhR4DT-1 and AhR3′DT-1 which were obtained from *Arachis hypogaea* (Figure 3). Such prenylated stilbenoids have shown very effective protective activities in human diseases as well [153]. Similar prenylated resveratrol compounds at the position 3′-, were also with the enzyme NovQ, obtained from *Streptomyces spheroids* [154,155]. Additionally, the enzymes CloQ and Orf2, obtained from *Streptomyces roseochromogeus* and *Streptomyces sp.* CL190, respectively were also reported by Botta and colleges as prenylation on resveratrol or related stilbenoids [155,156]. Another enzyme called NaphB that is obtained from *Streptomyces sp*. was also found to be responsible for the geranylation in resveratrol at the position 4- or both at the 4- and 2- positions [157]. Prenylated modified compounds are highly potent against many human diseases hence such a compound has high scope in the pharmaceutical aspect.

## 3. Conclusions

Because of its potent biological activities in terms of medical and nutraceutical values, resveratrol and its derivatives are of great interest and demand. Recently developed biological tools and techniques help to produce such a bioactive plant product in microbial hosts with low cost and minimal time range. Despite the lack of resveratrol biosynthetic genes in microbial hosts, they are a great alternative source for the large-scale production of resveratrol and its derivatives because of the application of metabolic engineering and synthetic biology approaches. Recent research has shown that a pure form of resveratrol can be produced in heterologously engineered microorganisms, which reduces extensive processing. However, still, the overall production of resveratrol and its derivatives is not adequate despite applying all the recent techniques. Therefore, each gene involved in the biosynthetic pathways should be well-optimized to improve enzyme activity in trans-located hosts by supplying sufficient precursors and regulating the concentration of resveratrol inside the cell, which could help to increase production. Therefore, there should be a focus on a combinatorial approach to directing every metabolite and precursor towards resveratrol production. Moreover, complete knowledge of synthetic and molecular biology of each component involved, such as the whole genome, transcriptome, proteome, and metabolome, during the biosynthesis of resveratrol help to increase the production from the microbial platform.

## Figures and Tables

**Table 1:** List of stilbene synthases identified and characterized from different plants.

Name	Source	Reference
VvSTS	*Vitis vinifera*	[50]
Pcu1STS	*Polygonum cuspidatum*	[51]
Pcu3STS	*Polygonum cuspidatum*	[51]
PsSTS	*Pinus strobus*	[52]
PdSTS	*Pinus densiflora*	[53]
AhSTS	*Arachis hypogaea*	[54]
PhSTS	*Parthenocissus henryana*	[55]
MaSTS	*Morus atropurpurea*	[56]
RtSTS	*Rheum tataricum*	[57]

**Table 2:** Microbial production of resveratrol and their derivatives.

Microbial Host	Incorporated Genes	Host Engineered	Substrate	Titer (mg/L)	References
***Escherichia coli*** **(Resveratrol)**					
*E. coli* BW27784	4CL(*A. thaliana*) STS (*A. hypogaea*)		*p*-Coumaric acid	0.16	[103]
*E. coli* BL21(DE3)	4CL (*N. tabacum*) STS (*V. vinifera*)		*p*-Coumaric acid	16	[68]
*E. coli* BL21(DE3)	Pal (*R. rubra*) 4CL (*L. erythrorhizon*) STS (*A. hypogaea*)		Tyrosine	37	[101]
*E. coli* BRB	4CL (*L. erythrorhizon*) STS (*A. hypogaea*) ACC (*C. glutamicum*) *F3H* and *FLS* (Citrus)		Cinnamic acid	155	[104]
			*p*-Coumaric acid	171	
*E. coli* BW27784	4CL (*A. thaliana*) STS (*A. hypogaea*)		*p*-Coumaric acid	105	[99]
*E. coli* BW27784	4CL (*A. thaliana*) STS (*A. hypogaea*)		*p*-Coumaric acid	404	[71]
	4CL (*A. thaliana*) STS (*V. vinífera*)			1380	
	4CL (*P. crispum*) STS (*A. hypogaea*)			142	
	4CL (*P. crispum*) STS (*V. vinífera*)			610	
	4CL (*A. thaliana*) STS (*V. vinífera*)		*p*-Coumaric acid and cerulenin	2340	[71]
*E. coli* C41 (DE3)	TAL (*S. espanaensis*) 4CL (*S. coelicolor*) STS (*A. hypogaea*)		*p*-Coumaric acid	104	[72]
*E. coli* BW25113 (DE3)	TAL (*R. glutinis*) 4CL (*P. crispum*) STS (*V. vinifera*)	Inactivation of *tyr*R and deletion of *trp*ED by chromosomal integration	Glucose	4.6	[77]
*E. coli* BW27784 (DE3)	4CL (*A. thaliana*) STS (*V. vinífera*)		*p*-Coumaric acid	1600	[105]
*E. coli* BL21 (DE3)	TAL (*R. glutinis*) 4CL (*P. crispum*) STS (*V. vinifera*) *matB* and *matC* (*R. trifolii*)		L-Tyrosine	35.02	[73]
*E. coli* BL21 (DE3)	4CL::STS, 4CL (*A. thaliana*)- STS (*A. hypogaea*) fusion enzyme		*p*-Coumaric acid	80.5	[100]
*E. coli* BL21 (DE3)	TAL (*T. cutaneum*) 4CL (*P. crispum*) STS (*V. vinifera*) *matB* and *matC* (*R. trifolii*) tyrA^fbr^ and aroG^fbr^ (*E. coli* K12)	Down-regulation of *fabD*, *fabH*, *fabB*, *fabF*, *fabI*	Glucose	304.5	[106]
*E. coli* BW25113	4CL2 (*P.crispum*) STS (*V. vinifera*)		*p*-Coumaric acid	268.2	[14]
*E. coli* C41 (DE3)	TAL (*S. espanaensis*) 4CL (*S. coelicolor*) STS (*A. hypogaea*)		Glucose	5.2	[95]
*E. coli* BL21(DE3)	TAL (*S. espanaensis*) 4CL (*A. thaliana*) STS (*A. hypogaea*)		Tyrosine	114.2	[102]
*E. coli* W(pheA^-^) Rg *E.coli* W-Vv	TAL (*R. glutinis*) tktA^fbr^ and aroG ^fbr^ (*E. coli*) 4CL (*S. coelicolor*) STS (*V. vinífera*)	Deletion of *phe*A	Glycerol	22.58	[49]
**Non- *E. coli* (Resveratrol)**					
*C. glutamicum* DelAro3	STS (*A. hypogaea*) 4CL (*P. crispum*)	Deletion of *phdB*, *pcaF* and *pobA*	*p*-Coumaric acid	12	[89]
			*p*-coumaric acid + cerulenin	158	
*C. glutamicum* DelAro3	TAL (*F. johnsoniae*) 4CL (*P. crispum*) STS (*A. hypogaea*) aroH (*E. coli*)	Deletion of *phdB*, *pcaF*, *qsuB* and *pobA*	Glucose	12	[90]
			Glucose+ cerulenin	59	
			Glucose(40 g/L)	4	
			Glucose(80 g/L)	12	
			Glucose (Fed-batch)	7	
*S. cerevisiae* W303-1A	4CL (*A. thaliana*) STS (*A. hypogaea*)		*p*-Coumaric acid	3.1	[70]
*S. cerevisiae* WAT11	4CL (*A. thaliana*):STS (*V. vinifera*)		*p*-Coumaric acid	5.25	[107]
*S. cerevisiae* WAT11	TAL (*R. sphaeroides*) 4CL::STS, 4CL (*A. thaliana*)-STS *V. vinifera*) fusionenzyme		Tyrosine	1.9	[108]
*S. cerevisiae* CEN. PK102-5B	TAL (*H. aurantiacus*) 4CL (*A. thaliana*) VST (*V. vinifera*)	Overexpression of *aro4*, *aro7*, and *acc1*	Glucose (fed-batch)	415.65	[82]
			Ethanol (fed-batch)	531.41	
*S. cerevisiae* ST4990	PAL (*A. thaliana*) C4H (*A. thaliana*) 4CL (*A. thaliana*) VST (*V. vinifera*) ACS (*S. enterica*) Overexpression of *atr2* (*A. thaliana*)	Overexpression of *aro4*, *aro7*, and *acc1* and deletion of *aro10*	Glucose (Fed-batch)	812	[69]
*S. cerevisiae* DHS2001	STS (*A. hypogaea*) 4CL (*S. coelicolor*)	Deletion of pks	*p*-Coumaric acid	0.4	[92]
*L.lactis*	TAL, 4CL, STS, ACC (different sources)		L-Tyrosine	0.45-1.37	[109]
Industrial Brazilian *S. cerevisiae* strain	STS (*V. vinifera*) 4CL (*A. thaliana*)		*p*-Coumaric acid	391	[83]
***Escherichia*** ***coli* (Resveratrol derivatives)**		**Substrate**	**Derivatives**		
*E. coli* C41 (DE3)	TAL *(S. espanaensis)* 4CL *(S. coelicolor)* STS *(A. hypogea)* Glycosyltransferase YjiC *(Bacillus spp.)*	Glucose	3-*O*-resveratrol β-D-glucoside	2.5	[110]
			4′-*O*-resveratrol β-D-glucoside	7.5	
*E. coli* BL21-CodonPlus (DE3)-RIPL	VvROMT (*V. riparia*)	Resveratrol	Pinostilbene	0.16	[111]
			Pterostilbene	0.04	
	SbROMT (*S. bicolor*)		Pinostilbene	34	
			Pterostilbene	0.16	
*E. coli* C41(DE3)	TAL (*S. espanaensis*) 4CL (*S. coelicolor*) STS (*A. hypogea*) SbOMT3, SbOMT1	Glucose	3,5-Dihydroxy-4′-methoxystilbene	2.50.2	[95]
			5-Hydroxy-3,4′-dimethoxystilbene		
*E. coli* W3110	4CL(*S.coelicolor* A3), STS (*V.vinifera*)	Cinnamic acid	Pinosylvin	34.89	[112]
	4CL(*S. coelicolor* A3), STS (*V. vinifera*) + reduced expression level of fabI gene	Cinnamic acid	Pinosylvin	52.67	
*E. coli* BW27784	4CL1(*A. thaliana*) STS(*A. hypogea*)	Caffieic acid	Piceatannol	13	[99]
*E. coli* BLR(DE3)	PAL(*Rhodotorula rubra*) 4CL(*Lithospermum* *erythrorhizon*) STS(*A. hypogea*) ACC(*Corynebacterium glutamicum*)	Phenylalanine	Pinosylvin	20	[104]
		Tyrosine	Pinostilbene	18	
			Pterositibene	5.8	
	PAL(*Rhodotorula rubra*) 4CL(*Lithospermum* *erythrorhizon*) STS(*A. hypogea*) ACC(*Corynebacterium glutamicum*) +OsPMT (*Oryza sativa*)	Phenylalanine	Pinosylvin monomethyether	27	
			+Pinosylvin dimethyl ether	27	
*E. coli* BL21(DE3)	PAL (*P. crispum*) 4CL(*S.coelicolor*) STS [*Pinus strobus* (two mutated T248A; Q361R)] Cerulenin	Glucose/phenylalanine	Pinosylvin	91	[91]
**Non-*E. coli* hosts (Resveratrol derivatives)**					
*C. glutamicum* DelAro_4_	4CL (*P. crispum*), STS (*A. hypogea*) OMT (*V. vinifera*):MalE (*E. coli*) metK (*E. coli*)	*p*-Coumaric acid	Pterostilbene	42	[113]
*S. cerevisiae* WAT11	4CL (*A. thaliana*), STS (*V. vinifera*) ROMT (*V. vinifera*)	Resveratrol	Pterostilbene	150	[114]
		*p*-Coumaric acid	Pterostilbene	2	
*S. cerevisiae* ST4993	*thaliana* (PAL, C4H, 4CL2) STS (*V. vinifera*) + feedback insensitive alleles of DHAP synthase (ScARO4K229L) and chorismate mutase (ScArO7G141S) + shikimate kinase aroL + deregulated variant of ACC1 + non-regulated version of acetyl-CoA synthase (ACS) + deletion of phenylpyruvate decarboxylase ARO10 + cytochrome P450 reductases ATR2 and CYB5 + SbROMT (*Sorghum bicolor*)	Glucose	Pterostilbene	5.5	[69]
*S. cerevisiae* ST4994	All the components of Strain ST4993 along with VvROMT(*V.vinifera*) instead of SbROMT		Pterostilbene	3.5	
